# Microbiota-constrained bacterial density limits phage infection in the gut and allows persistence of susceptible cells

**DOI:** 10.1016/j.isci.2026.117433

**Published:** 2026-10-06

**Authors:** Anouk Bertola, Nicolas Wenner, Leonardo Lemos Rocha, Timothy G. Keys, Médéric Diard

**Affiliations:** 1 Biozentrum, University of Basel, 4056 Basel, Switzerland; 2 Baxiva AG, Südstrasse 1, 8952 Schlieren, Switzerland

**Keywords:** bacteriophage, microbiota, *in vivo* evolution, gut colonization, phage resistance, *Escherichia coli*

## Abstract

With the rise of antibiotic resistance, bacteriophage-based treatments against bacterial infections have regained momentum. However, phage therapy has so far shown only moderate success in complex environments like the mammalian gut, where phages are particularly promising tools for microbiome engineering and specific elimination of opportunistic pathogens. Here, we use different murine models of intestinal colonization to quantitatively assess the minimal population size of susceptible *Escherichia coli* cells that is required for phage replication in the gut (replication threshold). We found that the composition of the intestinal microbiota is a key factor shaping phage–bacteria interactions by restricting the carrying capacity of susceptible host cells. We further demonstrate that the replication threshold enables coexistence of susceptible and resistant bacteria in phage-treated mice, with implications for phage-based applications in complex environments like the gut.

## Highlights

• The microbiota limits phage-susceptible bacteria below the phage replication threshold
• The phage replication threshold in the gut is 10^6^ bacteria/mL of intestinal content
• Phage-susceptible subpopulations of bacteria can persist below the replication threshold

## Graphical abstract

**Figure:**
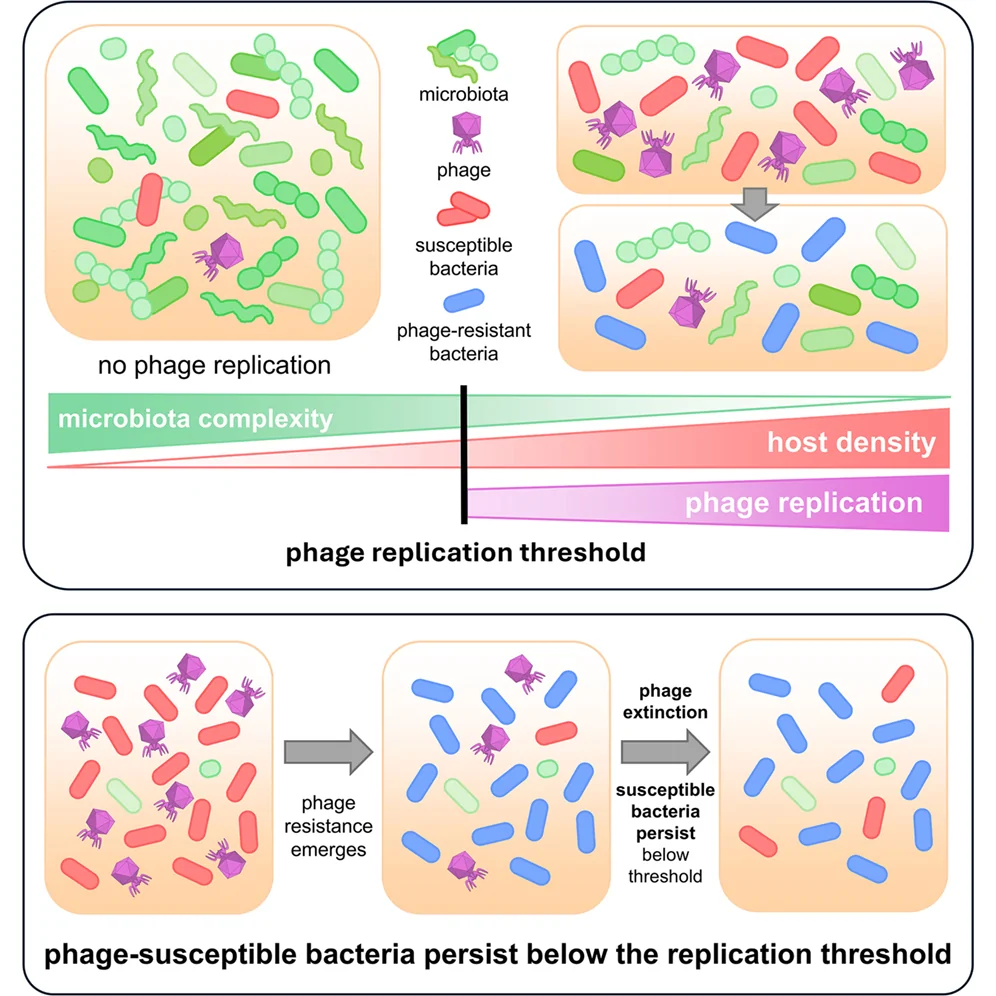

## Teaser

Microbiome; Viral microbiology

## Introduction

Bacteriophages (phages) are natural predators of bacteria and increasingly considered actionable alternatives to combat antibiotic-resistant pathogens.^1^ The ability of phages to infect a limited range of susceptible hosts allows specific targeting of bacterial strains within communities of bacteria. This could be particularly useful for modulating the composition of the intestinal microbiota, which plays a key role in human and animal health.^2^ In this context, where antibiotics disturb the microbiota and weaken the resistance to colonization by resistant pathogens,^3^ phages could allow precision microbiota engineering and elimination of potentially harmful bacteria.^4^ Multi-drug resistant “Enterobacteriaceae,” typically *Klebsiella* spp. and *Escherichia coli*, would be clinically relevant targets for therapeutic phages in the large intestine, which represents an important reservoir for these opportunistic pathogens.^5^^,^^6^

Many parameters influence the outcome of phage-bacteria interactions, including the phage adsorption rate, phage and bacterial densities, bacterial growth rate, and the evolvability of phage resistance and of the phage host-range.^7^^,^^8^ Polymicrobial communities tend to reduce the density of phage-susceptible cells and phages, which affects the ecological and evolutionary dynamics of phage-bacteria interactions.^9^ It has been shown that low concentrations of susceptible bacteria hinder phage infection, suggesting a phage replication threshold.^10^^,^^11^^,^^12^^,^^13^ All these factors were extensively studied in reductionist *in vitro* models; however, phage-bacteria interactions are much less understood and predictable in experimental infection models and in clinical trials where environmental parameters can play decisive roles.

Here, we focus on phage therapy in the gastro-intestinal tract. In this context, the impact of phages varies widely, ranging from significant reduction of bacterial loads to emergence of phage resistance and, sometimes, no discernible effect.^14^ This is because the gut is a particularly complex environment including the host immune system that interacts with both bacteria and phages,^15^^,^^16^ and a structure that can provide sanctuaries to bacteria or phages, facilitating coexistence.^17^ Moreover, bacterial gene expression in the gut can alter phage susceptibility when compared to *in vitro* conditions,^18^ and the physical properties of the intestinal content can influence the evolvability of the phage host-range.^19^ The cost of phage resistance in this environment can differ substantially from *in vitro*, hence driving different evolutionary outcomes.^20^^,^^21^ In order to improve the robustness of phage applications in complex environments such as the intestinal tract, it is therefore essential to understand the parameters that govern phage-bacteria interactions in this context.

This study investigates the phage replication threshold in the intestinal tract. Because the density of any invading bacterial species in the gut is highly dependent on the composition of the resident microbiota,^22^ we hypothesized that increasing the complexity of the gut microbiota should reduce the density and availability of phage-susceptible bacteria and thus impact phage replication and selection for resistance.

## Results

To assess phage-bacteria interactions, we worked with two phage-bacteria pairs. The two strains of *Escherichia coli* used, EcST73 and EcST1193, are multidrug-resistant strains from clinically relevant sequence types (ST73 and ST1193).^23^ EcST73 was isolated from a healthy traveler, and EcST1193 was isolated from a clinical case of neonatal sepsis,^24^ previously described as LU_100 in Larsson et al*.*^25^ The two strains produce different K-antigens (polysaccharide capsules) that are the receptors of two lytic phages, φYodit (using the K5 capsule produced by EcST73 as receptor) and φMoritz_NW-E1 (using the K1 capsule produced by EcST1193 as receptor).^25^ φYodit belongs to the genus *Kayfunavirus* and φMoritz to the genus *Vectrevirus*; both are *Autographivirales* with a short, non-contractile tail (Figure S1A), and both phages select for phage-resistant capsule mutants in their respective hosts *in vitro* (Figure S1B and Larsson et al.^25^). We selected capsule-specific phages because it is possible to enumerate phage resistant, capsule-defective mutants in a population according to their distinct translucent colony morphology on lysogeny broth (LB) agar plates without salt (Figure S1C).^25^

### The microbiota limits *E. coli* population size in the gut

The intestinal microbiota composition determines the *E. coli* carrying capacity of the host.^26^ Here, we used three different lines of mice harboring increasingly complex intestinal microbiota allowing modulation of the *E. coli* carrying capacity. In antibiotic-treated mice, the colonization resistance mediated by the microbiota is disrupted, allowing high loads of antibiotic-resistant strains.^27^ We also used conventional specific-pathogen-free (SPF) mice that harbor a complex microbiota, which confers strong colonization resistance against Enterobacteriaceae.^24^ As an intermediate model, we used gnotobiotic mice harboring a low-complexity microbiota (LCM) made up of only a few bacterial species and thus providing significant but reduced colonization resistance compared to SPF mice^22^(Figure 1A).

**Figure 1:**
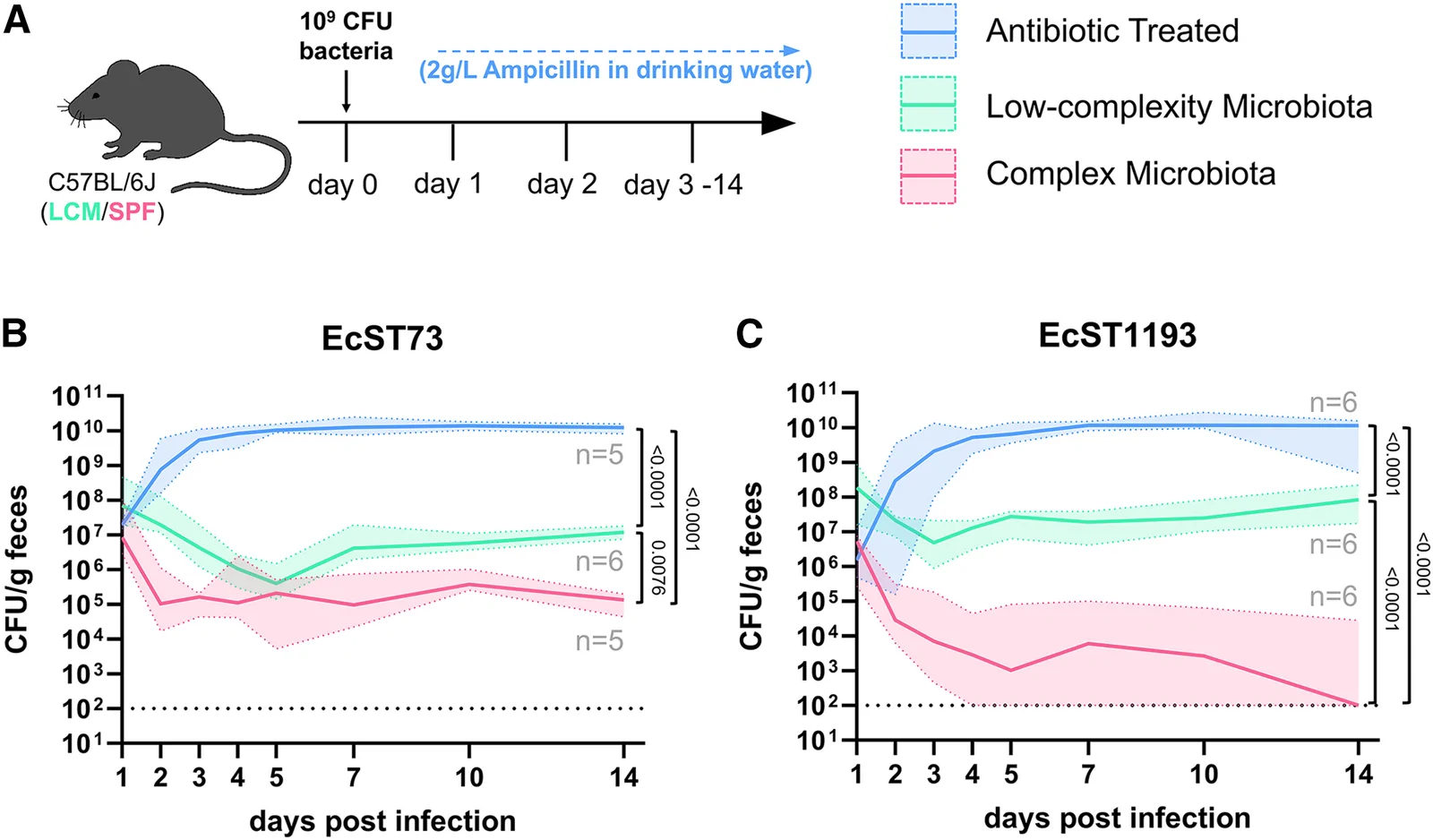
The complexity of the intestinal microbiota limits *E. coli* fecal population size (A–C) Experimental setup: in the antibiotic-treated group, conventional (SPF) mice were infected orally with 10^9^ bacteria, and antibiotic treatment started 24 h post infection. Low-complexity microbiota (LCM) and conventional (SPF) mice (complex microbiota) were infected orally with 10^9^ bacteria. Bacterial counts in fecal samples from mice infected with EcST73 (MDBZ1559) (B) and EcST1193 (MDBZ1913) (C). The dashed line indicates the detection limit. CFU, colony-forming units. Lines represent the median and the shaded area is the range. The number of animals in each group is indicated in gray. Number of independent cages: EcST73 SPF (3), EcST73 LCM (2), EcST73 ABX (2), EcST1193 SPF (4), EcST1193 LCM (2), and EcST1193 ABX (2). Statistical analysis was calculated using one-way ANOVA of log-normalized area under the curve (AUC) data, followed by Tukey’s multiple comparisons test. *p* values are reported.

When infecting the three models with either EcST73 (Figure 1B) or EcST1193 (Figure 1C), we observed a stabilization of the *E. coli* populations around model-specific levels. In SPF mice, EcST1193 was only maintained in 2/6 animals (3.1 × 10^4^ colony-forming units (CFU)/g feces, day 3–14 average for animals colonized until day 14), whereas colonization by EcST73 remained stable in all animals (3.1 × 10^5^ CFU/g feces, day 3–14 average). Both strains colonized antibiotic-treated mice at a high level (EcST73: 1.1 × 10^10^ CFU/g feces, EcST1193: 9.1 × 10^9^ CFU/g feces, day 3–14 average) and LCM mice at an intermediate level (EcST73: 5.8 × 10^6^ CFU/g feces, EcST1193: 3.4 × 10^7^ CFU/g feces, day 3–14 average). We also observed that the population sizes only stabilized 48 h after infection and thus administered phage treatment at that time point.

### Bacteriophage replication depends on the population density of susceptible bacteria

We then administered a single dose of 10^9^ plaque-forming units (PFU) phage φYodit or φMoritz to mice infected with EcST73 or EcST1193, respectively (Figure 2A). We observed no significant effect of the phages on the *E. coli* population sizes (Figures 2B, 2C, and S2). Phage titers varied between mouse models and between individual animals (Figures 2D and 2E). In most conventional mice, the phage was undetectable after 48 h, suggesting that the low population size of *E. coli* was not sufficient to sustain phage replication. While the bacterial population in SPF mice was more stable for EcST73 than for EcST1193, where only 4/7 mice were colonized at day 14 post infection, this was not sufficient to allow maintenance of φYodit. A similar dynamic was observed in uninfected mice treated with either phage φMoritz or φYodit (Figures S1D and S1E), suggesting no phage replication in conventional mice harboring a relatively small population of *E. coli*.

**Figure 2:**
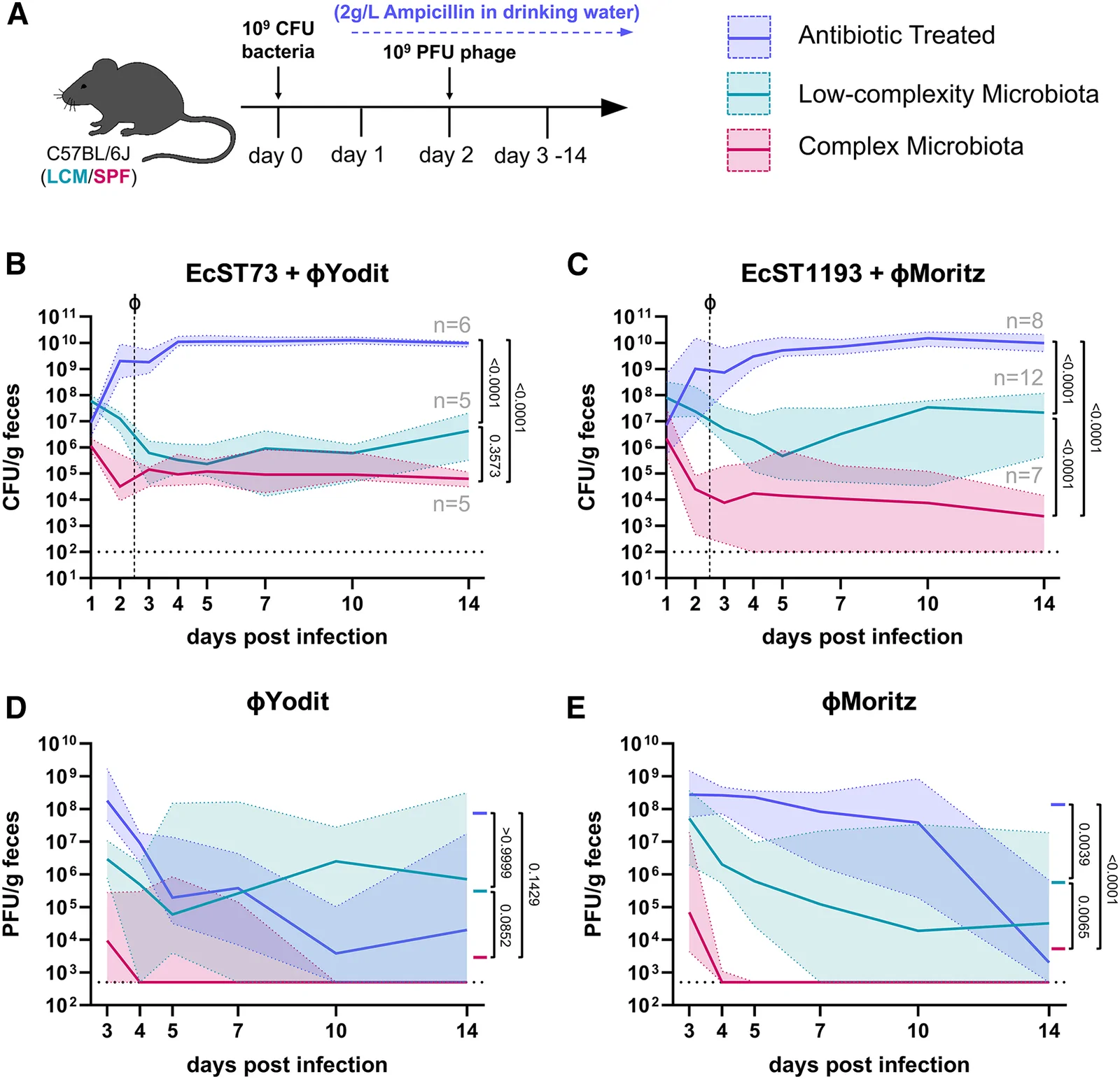
Complex microbial communities limit phage replication (A–E) Experimental setup: mice were infected orally with 10^9^ bacteria. After 24 h, the mice in the antibiotic treatment group received ampicillin in their drinking water. All mice were treated orally with 10^9^ phages 48 h after bacterial infection. Bacterial counts for mice infected with EcST73 (MDBZ1559) (B) and EcST1193 (MDBZ1913) (C) and treated with phage, as obtained from fecal samples. φYodit (D) or φMoritz (E) titers for mice infected with EcST73 and EcST1193. The dashed line is the detection limit. CFU, colony-forming units; PFU, plaque-forming units. Lines represent the median and the shaded area is the range. The number of animals in each group is indicated in gray. Number of independent cages: EcST73 SPF (3), EcST73 LCM (3), EcST73 ABX (2), EcST1193 SPF (3), EcST1193 LCM (5), and EcST1193 ABX (4). Statistical analysis was calculated using one-way ANOVA of log-normalized area under the curve (AUC) data, followed by Tukey’s multiple comparisons test. *p* values are reported.

In contrast, the phage populations were more stable with high phage titers for multiple days in both antibiotic-treated and LCM mice; however the phage titers eventually decreased in antibiotic-treated animals and some LCM mice (Figures 2D and 2E).

### Phage predation selects for resistance above a bacterial density threshold

The decrease in phage titer in mice where the overall *E. coli* population size sustained phage replication for few days could be due to a decrease in the frequency of susceptible hosts. With the receptor for both phages being the capsule, we were able to track the frequency of phage-resistant, capsule-defective mutants in feces samples according to colony morphology on salt-free LB agar plates (Figure S1C).

In the presence of phages, we observed a rapid increase of capsule-defective mutants in antibiotic-treated mice in both bacterial strains (Figures 3A and 3B). In the absence of phage, no capsule-defective mutants were observed (Figures S1F and S1G). Twenty-four hours after phage administration, the capsule-defective mutants made up all detected EcST73 clones, and phage titers subsequently decreased. In EcST1193, the fixation of capsule mutants was delayed, and phage titers remained higher for a longer period. Low phage titers correlated with the diminishing capsulated population, suggesting reduced phage replication due to declining density of susceptible hosts. Even though we did not detect capsulated bacteria after two weeks, the phage did not go extinct in all animals, suggesting that there may still be a large enough subpopulation of susceptible cells sustaining phage replication. In contrast, we did not observe any capsule-defective mutants in conventional SPF mice that received phages. Lack of successful phage replication at these low bacterial densities failed to put pressure on the bacteria to evolve phage resistance.

**Figure 3:**
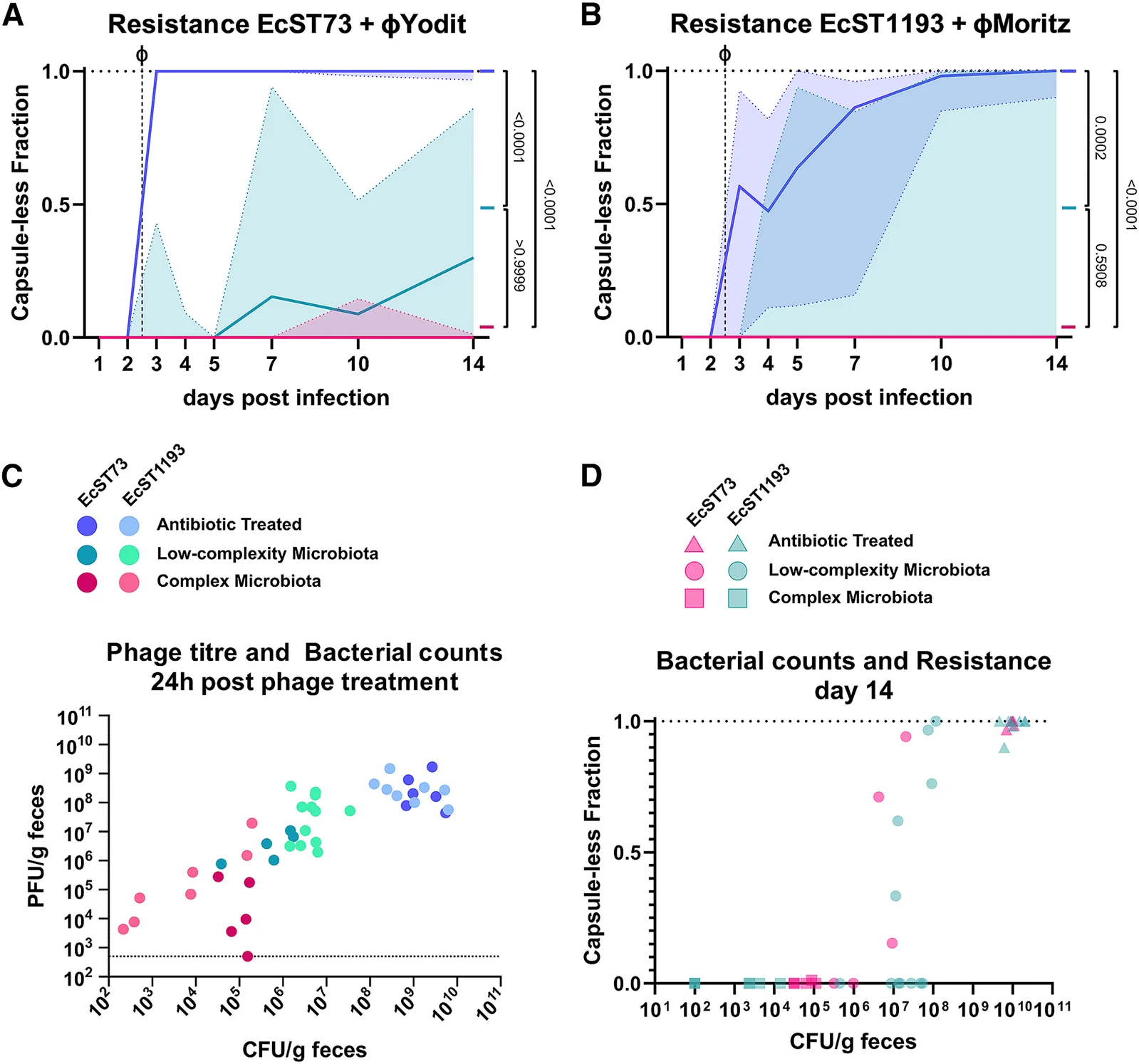
Effective phage replication above a threshold of 10^6^ susceptible bacteria per gram of feces (A–D) Fraction of capsule-less cells from mice infected with EcST73 (MDBZ1559) (A) and EcST1193 (MDBZ1913) (B) and treated with phage, as obtained from fecal samples. The line shows the median and the shaded area represents the range, n = 5–13. The detection limit is 0.01. (C) Comparison of phage titers and bacterial counts 24 h after phage treatment (day 3 post-infection) (D) Comparison of translucent fraction and colonization level of phage-treated mice for both bacterial strains at day 14 post-infection. CFU, colony-forming unit; PFU, plaque-forming units. Statistical analysis was calculated using one-way ANOVA of area under the curve (AUC) data, followed by Tukey’s multiple comparisons test. *p* values are reported.

In LCM mice, the evolutionary trajectories varied greatly between animals. In some, the phage disappeared without capsule defective mutants arising, whereas in others, the phage-resistant bacteria made up a large fraction of the population and the phage went extinct. This large variation between individuals suggested that the average bacterial population size in LCM mice was close to a threshold below which phage replication was not sustained and that inter-individual variations can have a strong effect. Initial phage titers in LCM mice were higher than in SPF mice, suggesting phage replication took place at least in the beginning but was not always enough to drive the emergence of resistance.

Sequencing of phage-resistant bacteria after 14 days of evolution in antibiotic-treated mice revealed mutations in genes involved in capsule synthesis, transport and regulation (Table S1). In both EcST73 and EcST1193, we observed mutations in the transcriptional regulator *emrR*, also known as *mprA*, involved in regulation of capsule production, with *emrR* mutants lacking a capsule.^21^^,^^25^ We also observed mutations in and upstream of the capsule biosynthesis gene *kpsM*, a gene that is required for capsule polysaccharide transport.^28^ In some clones of EcST1193, we observed mutations in *neuS* and *neuE*, which are involved in polysialic acid biosynthesis, required for formation of the K1 capsule.^29^ In addition to the mutations conferring the phage resistance, most clones also acquired mutation in *malF*, *malT*, or *malI*, genes involved in maltose and maltodextrin uptake and metabolism. These mutations are typical for *E. coli* evolving in the gut of antibiotic-treated mice.^30^

For both phage-bacteria pairs, phage titers were proportional to bacterial levels 24 h after phage administration (Figure 3C). However, when comparing both strains’ population sizes with the emergence of capsule-less mutants at day 14 post infection, we observed no capsule-less mutants below a population size of 10^6^ CFU/g feces (Figure 3D). Below this threshold, phages cannot replicate, and there is no selective pressure against the capsule.

### A subpopulation of susceptible cells persists below the phage replication threshold

Next, we hypothesized that a population of susceptible bacteria—several orders of magnitude smaller than the population of capsule-defective mutants—could survive below the phage replication threshold. Such susceptible populations cannot be detected with our phenotypic readout to assess the frequency of resistance, i.e., the number of translucent colonies on plates. To reveal this subpopulation, we infected antibiotic-treated (SPF) mice with a mixture of antibiotic-tagged EcST73 wild-type (WT) bacteria (WT) and EcST73 Δ*kps*, a phage-resistant capsule-knockout mutant quantifiable on antibiotic-containing selective media (Figure 4A).

**Figure 4:**
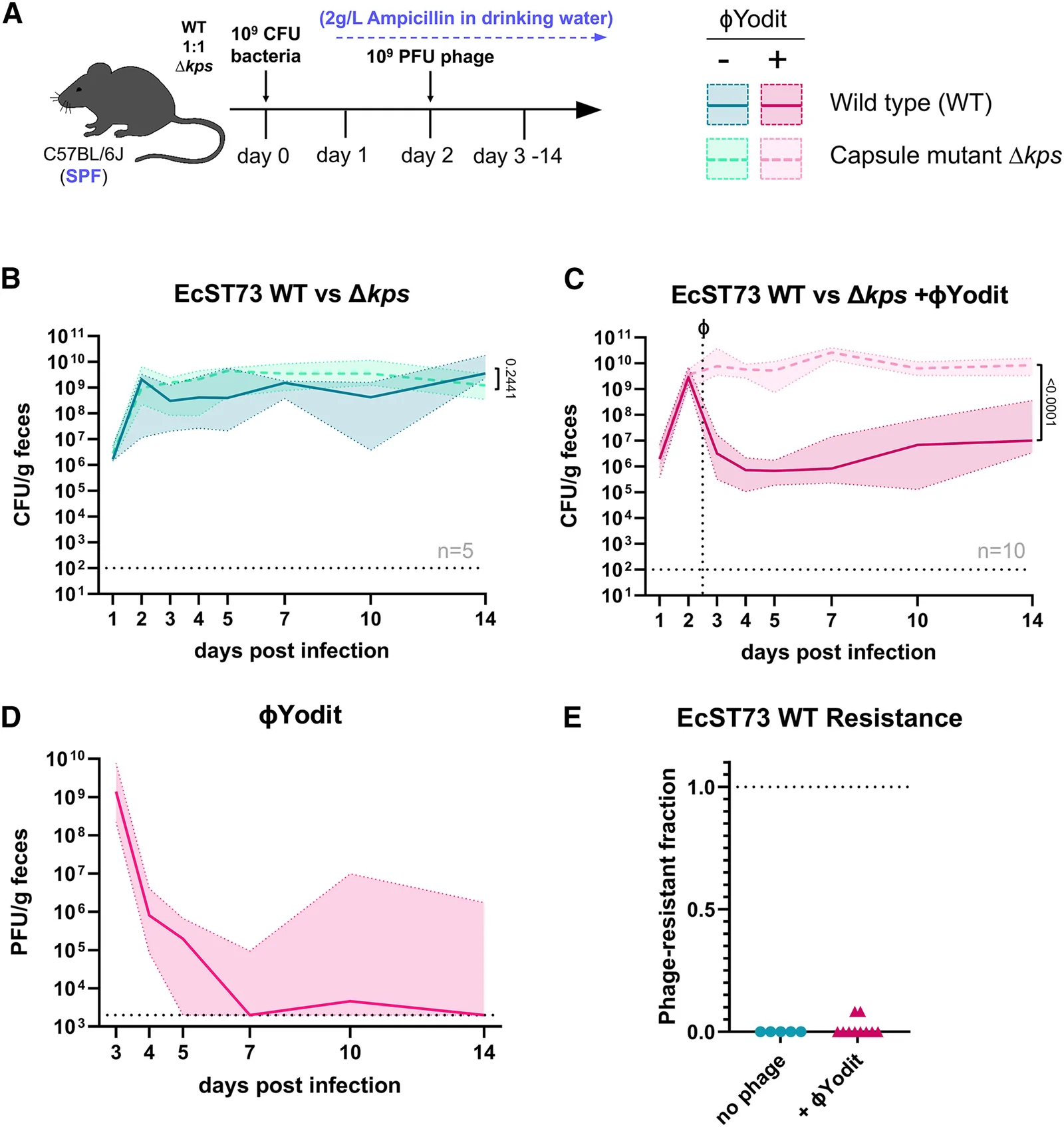
Competition between WT and Δ*kps* strains in antibiotic-treated SPF mice reveals the population size of persistent susceptible cells in presence of phages (A–D) Experimental setup: SPF mice were infected orally with 10^9^ bacteria (EcST73 WT [MDBZ1915] and EcST73 Δ*kps* [MDBZ1912] in a 1:1 ratio, tagged with distinct antibiotic-resistance cassettes). After 24 h, the mice received ampicillin in their drinking water. Mice in the phage treatment group were treated orally with 10^9^ phages 48 h after infection. Bacterial counts of EcST73 in absence (B) and presence (C) of phage treatment. Samples were plated on salt-free LB agar plates containing selective antibiotics to differentiate the WT and Δ*kps* strains. Phage titers of φYodit (D) as enumerated from fecal samples. The line shows the median and the shaded area represents the range. The number of animals in each group is indicated in gray. Number of independent cages: EcST73 ABX, no phage (2), and EcST73 ABX + phage (4). Statistical analysis was calculated using one-way ANOVA of log-normalized area under the curve (AUC) data, followed by Tukey’s multiple comparisons test. *p* values are reported. CFU, colony-forming unit; PFU, plaque-forming units. (E) Testing of the “WT” population for phage susceptibility using a mini-plaque overlay assay. 24 clones were tested per animal.

In mice that were not exposed to any phage, these two strains coexisted at similar and stable levels (Figure 4B). Upon exposure to phage φYodit, the EcST73 WT fraction of the population decreased by several orders of magnitude, as the susceptible cells were killed by the phage. The EcST73 WT population then stably coexisted with the phage-resistant EcST73 Δ*kps* population around a density of 2.4 × 10^6^ CFU/g feces (average CFU/g feces, days 3–7 p.i., Figure 4C). This level was similar to the density observed in mono-colonized LCM mice, at the limit of what was required to sustain phage replication. This is in line with the extinction of the phage in 6/10 animals, clearly showing that this remaining susceptible population was close to the phage replication threshold (Figure 4D). After extinction of the phage, the density of WT cells increased in some animals, suggesting that the remaining susceptible population could recover in numbers. Notably, most EcST73 WT cells were still phage-susceptible after 10 days (Figures 4E and S3).

## Discussion

To replicate, lytic phages must reach bacterial hosts in a permissive physiological state,^31^ which is not obvious in a complex environment like the intestinal tract. Here, we hypothesized that phage replication should be particularly constrained when the microbiota limits the carrying capacity of phage hosts. To quantitatively address the impact of the microbiota on the outcome of phage-bacteria interactions, we varied the complexity of the microbiota composition in mice, thereby modulating the *E. coli* carrying capacity of the gut (Figure 1). We made two important observations with this approach.

First, phages are not able to replicate and go extinct when the gut microbiota limits the population size of genetically susceptible *E. coli* below a certain threshold (Figures 2 and 3). We found that the population size of *E. coli* necessary to sustain phage replication, hence driving selection for resistance, is 10^6^ bacteria/g of feces. This corresponds to 1.3×10^6^ bacteria/mL of cecal content, assuming 400 mg of cecal content/cecum in 300 μL,^32^ which is a hundred times higher than the “proliferation threshold” previously determined *in vitro* with multiple phage-bacterium pairs, including phage T4 and *E. coli* (10^4^ bacteria/mL).^13^ The proliferation threshold is the minimum density of susceptible bacteria required to sustain a phage population, where the rate of new phage production exceeds the rate of phage loss. It is also dependent on the phage adsorption rate,^12^ which in turn is influenced by the environment.^33^ All these factors would differ in the gut, when compared to *in vitro* conditions in liquid culture. The diffusion rate of phage particles in the gut will be hindered by the viscosity of the medium and the structure, which creates micro-environments and inert obstacles, as well as off-target adsorption of phage particles.^33^ The removal of phage particles in the gut is influenced by the flow of intestinal content, phage uptake by epithelial cells,^34^ and inactivation by digestive enzymes or bile salts.^35^ Although mucus-adhesion can be beneficial for phage retention in the gut, potentially reducing the loss of free virions,^36^^,^^37^ susceptible cells can be difficult to reach by phages in such a complex environment,^17^ thereby increasing the chance for phages to be cleared before infection. Additionally, the population size detected in feces does not necessarily reflect the fraction of cells actively growing in the gut that is the effective population size of hosts that could sustain phage replication. Moreover, some growing cells may not express the phage receptor,^18^ in this case the capsule, that is indeed often heterogeneously expressed in Enterobacteriaceae.^38^

The second important finding is that the complex microbiota is not essential to generate conditions that protect susceptible cells against phages in the gut. A high density of phage-resistant mutants is sufficient (Figure 4). In the absence of microbiota but in presence of a high density of resistant cells, susceptible *E. coli* cells persisted in the gut at a population size close to the threshold of 10^6^ bacteria/g of feces. This experiment also demonstrates that a concentration of phages orders of magnitude higher than susceptible cells (2 × 10^9^ phages for 5 × 10^6^ bacteria/g of feces at day 3 p.i., Figure 4) is not sufficient to eliminate this population. Flooding a population of susceptible cells with phages, hence driving targets’ extinction without relying on secondary phage infections (i.e., passive therapy^10^) is therefore unlikely to eliminate susceptible cells from the gut. The characteristics of this remaining susceptible population, as well as the niche in which it resides, remain to be investigated.

Our observations reveal that the proliferation threshold could limit applications aiming at engineering the intestinal microbiota with phages. If one considers targeting antibiotic-resistant Enterobacteriaceae and chooses *E. coli* as a representative species in humans, the estimated intestinal loads vary between 10^2^ and 10^9^ CFU of *E. coli*/g of feces.^39^^,^^40^ Our results suggests that phages should not replicate in patients carrying *E. coli* at concentration below 10^6^ bacteria/g of feces during treatment. Moreover, although phages could significantly reduce a population of *E. coli* initially high enough, they should fail at driving extinction of susceptible cells. This has been observed in mice infected by high loads of *E. coli* LF82^41^ or *Klebsiella pneumoniae*^42^ and treated by cocktails of phages. Similar results were observed in experiments performed in calves using phages against enteropathogenic *E. coli* (EPEC).^43^ Moreover, phages did not coexist with susceptible cells surviving below the replication threshold, instead the phage counts decreased below the detection limit in most cases, allowing targets of phage therapy to regrow (Figure 4C). Achieving complete decolonization requires increased understanding of the conditions that allow a low but stable population of susceptible cells to escape phages in the intestinal tract. For many diseases, even a transient reduction of bacterial levels can be sufficient to improve outcome and reduce mortality, as shown in multiple animal studies.^41^^,^^42^^,^^44^ In human patients who suffer from intestinal infections with Enterobacteriaceae like *E. coli*, there is often a disturbance of the microbiota, be it from antibiotic use^5^ or dysbiosis caused by the pathogen.^45^ In these cases, the density of target bacteria might be high enough for phage treatment to succeed.

### Limitations of the study

The number of bacterial strains and phages in our investigation remains limited, and only a single dose of phage was used. In models with low phage replication, the repeated administration of higher phage doses could increase the probability of phage infection. Nevertheless, in situations where the bacterial density is too low to support phage replication, we do not expect any effects of additional phage administration. Both our phages are capsule-specific *Autographivirales,* and these phage-specific properties could affect the replication threshold. However, others have shown effects of lytic phages in the gut down to similar bacterial densities, with diverse phages depending on other receptors for infection.^42^^,^^46^ This suggests that the replication threshold, quantified in this study, might be a general feature of phage-bacteria interaction in the intestinal context.

## Resource availability

### Lead contact

Requests for further information and resources should be directed to and will be fulfilled by the lead contact, Médéric Diard (mederic.diard@unibas.ch).

### Materials availability

Bacterial strains and bacteriophages are available upon request with a materials transfer agreement (MTA).

### Data and code availability

• Phage and bacteria genomes have been deposited at GenBank and are publicly available as of the date of publication under the following accession nos. ϕMoritz (Genbank: PQ476011, PhiNW-E1), ϕYodit (Genbank: PZ544513), EcST73 MDBZ1559 (Genbank: PRJNA1480670), and EcST1193 MDBZ1340 (Genbank: PRJNA1502767).
• Raw sequencing data have been deposited at GenBank and are publicly available in BioProject Genbank: PRJNA1502767 as of the date of publication.
• Raw data from all figures as well as information on individual experiments and animals were deposited on Mendeley Data: https://doi.org/10.17632/289n3kxzdg.1.
• This paper does not report original code.
• Any additional information required to reanalyze the data reported in this paper is available from the lead contact upon request.

## Acknowledgments

We would like to thank the members of the Diard research team for support and advice as well as the staff of the Biozentrum and of the animal facilities in Basel. This project was funded by 10.13039/501100001711SNSF professorships # PP00P3_176954 and PP00P3_213978 and the ERC Consolidator grant ECOSTRAT #101002643 allocated to M.D. and supporting N.W. and L.L.R. A.B. was supported by the University of Basel and T.G.K. by an Innosuisse grant (117.143 IP-LS).

## Author contributions

A.B. and M.D. conceptualized the study and designed the experiments. A.B. performed the experiments and analyzed the results. A.B. and M.D. wrote the manuscript. N.W. and L.L.R. constructed bacterial strains and provided technical support. T.G.K. identified the capsule type of EcST73 and EcST1193.

## Declaration of interests

The authors declare no competing interests.

## STAR★Methods

### Key resources table

**Table:** 

REAGENT or RESOURCE	SOURCE	IDENTIFIER
**Bacterial and virus strains**		

EcST73	This work	MDBZ1559, Genbank:PRJNA1480670
EcST1193 (LU_100)	Larsson et al.^25^	MDBZ1340, Genbank:PRJNA1480995
EcST1193 [Tn7:*aac3_IV* Apra^R^ tagged]	This work	MDBZ1913
EcST73 Cm^R^ [Tn7:*cat*, carries mutations in *cdiA* and gene 4325]	This work	MDBZ1915
EcST73 Δ*kps* [Δ*kps*::*aphT*]	This work	MDBZ1912
ϕYodit	This work	MDPC0052, Genbank:PZ544513
ϕMoritz (ϕNW-E1)	Larsson et al.^25^	MDPC0031, Genbank:PQ476011
Raw sequencing data	This work	Genbank:PRJNA1502767

**Chemicals, peptides, and recombinant proteins**		

Ampicillin Sodium salt (Amp)	PanReac AppliChem	Cat#A0839
Kanamycin Sulfate (Kan)	PanReac AppliChem	Cat#A1493
Chloramphenicol (Cm)	PanReac AppliChem	Cat#A1806
Apramycin sulfate salt	Sigma-Aldrich	Cat#A2024
GoTaq® G2 Green Master Mix	Promega	Cat#M7822
Phusion™ High-Fidelity DNA Polymerase	ThermoFisher Scientific	Cat#F530S
dNTPs mix (10 mM each)	Hangzhou Bioer Technology co	Cat#BSA14S2B
DNAse I	Norgen	Cat#25732
RNAse A	Roche	Cat#10109169001

**Critical Commercial Assays**		

Phage DNA Isolation Kit	Norgen	Cat#46800
Quick-DNA Miniprep Plus Kit	Zymo Research	Cat#D4069
NucleoSpin® Gel and PCR clean up kit	Macherey-Nagel	Cat#740609.250
MinION or GridION Flow Cell R10 Version	Oxford Nanopore Technologies	Cat#FLO-MIN114
Rapid Barcoding Kit 24 V14	Oxford Nanopore Technologies	Cat#SQK-RBK114.24

**Deposited Data**		

Raw data for all experiments & mouse sex & cohousing information	This Study	https://doi.org/10.17632/289n3kxzdg.1

**Experimental models: Organisms/strains**		

Mouse: C57BL/6J Specific-pathogen-free	Bred inhouse	87 - C57BL/6J
Mouse: C57BL/6J Low-Complexity Microbiota	Bred inhouse	2068 – B6 LCM

**Oligonucleotides**		

EcST73 capsule knockout primer forward ggtagcgtacctgaagagattaggatca catcatcaaatgCATATGAATATCCTCCTTAG	This Study	OMD23_168
EcST73 capsule knockout primer reverse tgtgactgtggcattatttccgtgcaaagga gctgatatgGTGTAGGCTGGAGCTGCTTC	This Study	OMD23_169
EcST73 check for capsule knockout primer forward ATAAGCATGGACTGACCATGG	This Study	OMD21_761
EcST73 check for capsule knockout primer reverse CCTGCGGAAATAATTTCTGCTG	This Study	OMD21_762

**Recombinant DNA**		

pSIM5 - Temperature-sensitive plasmid carrying temperature-inducible λ-red recombination	Datta et al.^47^	pSIM5
pGRG36-*cat* - Temperature-sensitive plasmid for Tn7-based antibiotic tagging	Lentsch et al.^48^	pGRG36-*cat*
pGRG36-*aac3_VI* - Temperature-sensitive plasmid for Tn7-based antibiotic tagging	Lentsch et al.^48^	pGRG36-*aac3_VI*
pKD4 – *frt-aphT-frt* template plasmid	Datsenko et al.^49^	pKD4

**Software and algorithms**		

Flye	Kolmogorov et al.^50^	https://github.com/mikolmogorov/Flye
Pharokka	Bouras et al.^51^	https://github.com/gbouras13/pharokka
kTYPer	Miravet-Verde et al.^52^	https://github.com/SushiLab/kTYPr
GraphPad Prism 10 for Windows	GraphPad Software LLC	Version 10.2.0 (392)

### Experimental model and study participants details

All animal experiments were performed according to Swiss legislation and following the 3R principle and were approved by the cantonal veterinary authority (Kantonales Veterinäramt Basel-Stadt) under licence 2987_36493. Conventional SPF C57BL/6J mice or C57BL/6J mice carrying a low-complexity microbiota (LCM; adapted from altered Schäldler-Flora^53^) were used. Eight-to fourteen-week-old mice were used for all experiments, males and females were used indiscriminately as sex had no effect on colonisation outcomes. Colonisation with EcST73 and EcST1193 was not pathogenic and did not cause any visible symptoms.

### Method details

#### Culture conditions

Bacterial strains were grown on LB (10 g/L Tryptone, 5 g/L yeast extract, 10 g/L NaCl) or salt-free LB (10 g/L Tryptone, 5 g/L yeast extract) agar plates (1.5% Agar) with the appropriate antibiotics (ampicillin 100 μg/mL, chloramphenicol [Cm] 15 μg/mL, kanamycin [Kan] 50 μg/mL or apramycin 50 μg/mL) with overnight (16–20 h) incubation at 37°C. For liquid cultures, 2 mL of LB were inoculated with a single colony and grown overnight or for 4 h at 37°C with shaking at 200 rpm.

#### Plaque assay for phage quantification

For enumerating infectious phage particles, a top agar plaque assay was performed. A 12 cm × 12 cm square Petri dish or a 8.6 cm diameter round Petri dish (Sarstedt) containing LB Agar (1.5%) was overlaid with 0.5% agar LB (Top Agar) inoculated with bacterial culture. For square plates, 10 mL of warm (50°C) top agar were inoculated with 250 μL of dense bacterial culture and for round plates, 4 mL of top agar were inoculated with 100 μL of dense bacterial culture. Phage lysates were diluted in LB in 10-fold dilutions and 5 μL was spotted on top of the top agar and allowed to air-dry for 10–30 min. Plates where then incubated overnight at 37°C. Phage titers were calculated from the visible plaques after incubation.

#### Phage isolation

The K1-specific phage ϕMoritz (ϕNW-E1) was previously described in Larsson et al.^25^ and the genome is accessible on GenBank: PQ476011.1.

The K5-specific phage ϕYodit, that targets EcST73, was isolated from pooled waste water inlet (24h) from the ARA Basel wastewater treatment plant. 50 mL of waste water was filtered using a 0.2 μm filter, ZnCl_2_ was added to a concentration of 20 mM and incubated for 1 h at 37°C. The precipitates were pelleted by centrifugation and resuspended in 1 mL of LB. Of this, 300 μL was added directly into 10 mL top agar containing EcST73, poured onto a square LB agar plate and incubated overnight at 37°C. The next day, isolated plaques were re-suspended in 200 μL of LB and 10*x* dilutions were spotted on a new lawn of EcST73, and this was repeated twice to ensure clonal phage isolation.

To obtain high-titre lysates of ϕMoritz and ϕYodit, an exponential-phase bacterial culture of the suitable host (in 10 mL LB) was infected with 10 μL (∼10^7^ PFU) of phage lysate and incubated overnight at 37°C with shaking at 200 rpm. In parallel, a top agar plaque assay with 250 μL dense bacterial culture and ∼10^3^–10^4^ PFU was inoculated directly into the top agar on a square LB agar plate and inoculated overnight at 37°C. After incubation, the top agar was scraped into the liquid culture, incubated at RT on the shaking table and then spun down by centrifugation for 20 min at 4500 rpm. The resulting lysate was filtered using 0.2 μm filters and phage titers were determined using plaque assays.

#### Bacterial genome editing

Deletion mutants of the capsule biosynthetic gene cluster (Δkps = kpsM*, kpsT, EAPBFINA_00824, EAPBFINA_00825, EAPBFINA_00827, EAPBFINA_00827, ugd_1, kpsS, kpsC, kpsU, kpsD, kpsE, kpsF*) in EcST73 was constructed by *λ red* recombination^49^^,^^54^ using helper plasmid pSIM5 and template plasmid pKD4.

Electrocompetent cells carrying pSIM5 were prepared from bacteria grown to exponential phase in salt-free LB at 30°C. To induce the *λ red* genes, the cultures were incubated at 42°C for 45 min with shaking and the bacteria were washed twice with cold sterile MilliQ water (ddH_2_0). Finally, the electrocompetent bacteria were concentrated 100*x* in ddH_2_0.

Primers containing 40-nucleotides homologous regions flanking genes of interest were used to amplify the Kan^R^ cassette (*frt-aphT-frt*) from pKD4, using Phusion DNA polymerase. The PCR products were analyzed by gel electrophoresis and purified using a NucleoSpin Gel and PCR Clean-up kit (Macherey-Nagel). Induced electrocompetent cells carrying pSIM5 were transformed with the PCR products (1–2 μg). After recovery in 2 mL of SOC media (for 2 h or overnight at 37°C 200 rpm), bacteria were plated on LB + Kan plates, and incubated overnight at 37°C. The temperature-sensitive plasmid pSIM5 was eliminated by an overnight passage at 42°C.

The deletions were confirmed by colony PCR, using GoTaq Polymerase and external primers. The obtained PCR fragments were Sanger-sequenced by Microsynth AG (Balgach, Switzerland).

Tn7-based antibiotic tagging using pGRG36^55^ derivative plasmids was performed as described in Lentsch et al.^48^

#### Murine models of intestinal colonisation

Mice were infected with 10^9^ CFU bacteria in 100 μL Phosphate-buffered solution (PBS) by oral gavage on day 0. For the antibiotic-treated mouse model, the drinking water was replaced with autoclaved tap water containing 2 g/L of Ampicillin 24h after infection (on day 1). In the phage treatment groups, 10^9^ PFU of phage in exhausted lysogeny broth (LB) were administered by oral gavage. On days 1–5, 7, 10 and 14, fecal samples were collected and processed as described below.

#### Determining phage and bacterial load from fecal samples

Fecal pellets collected freshly from mice were weighed and homogenized in 1 mL PBS +10% Glycerol. Dilutions were plated on selective salt-free LB plates and incubated overnight at 37°C. Colonies were counted and bacterial counts were calculated per gram of feces. When antibiotic-tagged strains were used in competition experiments (Figure 4), fecal samples were plated on salt-free LB plates containing the corresponding antibiotics (Chloramphenicol 15 μg/mL, Kanamycin, 50 μg/mL).

For phage titer enumeration, 200 μL of homogenized fecal pellet in PBS+glycerol was treated with 25 μL of chloroform. A top agar plaque assay was then prepared by solidifying 10 mL of top agar with 250 μL dense bacterial culture of a suitable host (EcST73 or EcST1193) in a square Petri dish. Dilutions of phage lysate were spotted on top of the top agar and after drying the plates were incubated overnight at 37°C. Plaques were counted and phage titer was calculated per gram of feces.

#### 24-Well plaque assay to determine phage susceptibility

To confirm phage resistance, a 24-well mini plaque assay was performed. Per animal, 24 bacterial clones were picked from feces plated on selective salt-free LB plates and inoculated into 50 μL of LB in a 96-well plate and incubated 2h at 37°C, 200 rpm. The cultures were then resuspended in 250 μL of warm top agar and added into a well of a 24-well tissue culture plate on top of a base of 500 μL of LB agar. After the top agar solidified, 10 μL of phage lysate containing ∼10^1^–10^2^ PFU was spotted in each well. The plates were incubated overnight at 37°C. Phage sensitivity was readout by observing the presence/absence of plaques in each well. Wells that showed no plaques were re-tested to confirm phage resistance.

#### Genome sequencing

Phage DNA was isolated using a Phage DNA isolation kit (Norgen biotek corp. cat.: 46800) with additional DNAse and RNAse treatment. Bacterial DNA was isolated from overnight cultures using a Quick-DNA Miniprep Plus Kit (Zymo Research, cat. No D4069).

Sequencing was performed inhouse using Oxford Nanopore MinION long-read sequencing technology with 75 ng of purified DNA using the MinION Flow Cells R10 (ref. 10.4.(1) and Rapid Barcoding Kit 24 V14 (Oxford Nanopore). Base calling was performed using Guppy v6.5.7+ca6d6af, and the base calling model was dna_r10.4.1_e8.2_400bps_sup (Oxford Nanopore).

Phage genomes were assembled using flye^50^ and annotated with Pharokka^51^ using Galaxy^56^ servers. Bacterial genomes were analyzed using the Evo-genome-analysis pipeline (https://github.com/mmolari/evo-genome-analysis), as described in Wenner et al*.*^19^ Minimum frequencies for detection of different mutation types were *clips* > 0.15, *consensus* > 0.9, *gaps* > 0.9 and *insertions* > 0.3.

#### Determination of capsular serotype (K-type)

*In silico* capsular serotyping was performed using kTYPr (https://github.com/SushiLab/kTYPr) with default parameters.^52^ Capsule gene clusters were compared to reference clusters using the integrated clinker output option.

### Quantification and statistical analysis

Time-course experiments were compared by performing One-way ANOVA on the area under the Curve of LOG_10_-normalised data, followed by Tukey’s multiple comparisons test. All statistical analyses were done in GraphPad Prism 10. *p*-values, n = number of animals and detection limits are reported directly in figures.
