# Supplementary material for: Microbiota-constrained bacterial density limits phage infection in the gut and allows persistence of susceptible cells
Source: iScience. 2026 Oct 6;29(11):117433. doi: 10.1016/j.isci.2026.117433 (PMC13670935; doi:10.1016/j.isci.2026.117433)
Supplement: Supplementary file 1 [file mmc1.pdf]

iScience, Volume *29*

Supplemental information

Microbiota-constrained bacterial density limits phage infection in the gut and allows persistence of susceptible cells

Anouk Bertola, Nicolas Wenner, Leonardo Lemos Rocha, Timothy G. Keys, and Médéric Diard

Supplementary Information

Supplementary Figures

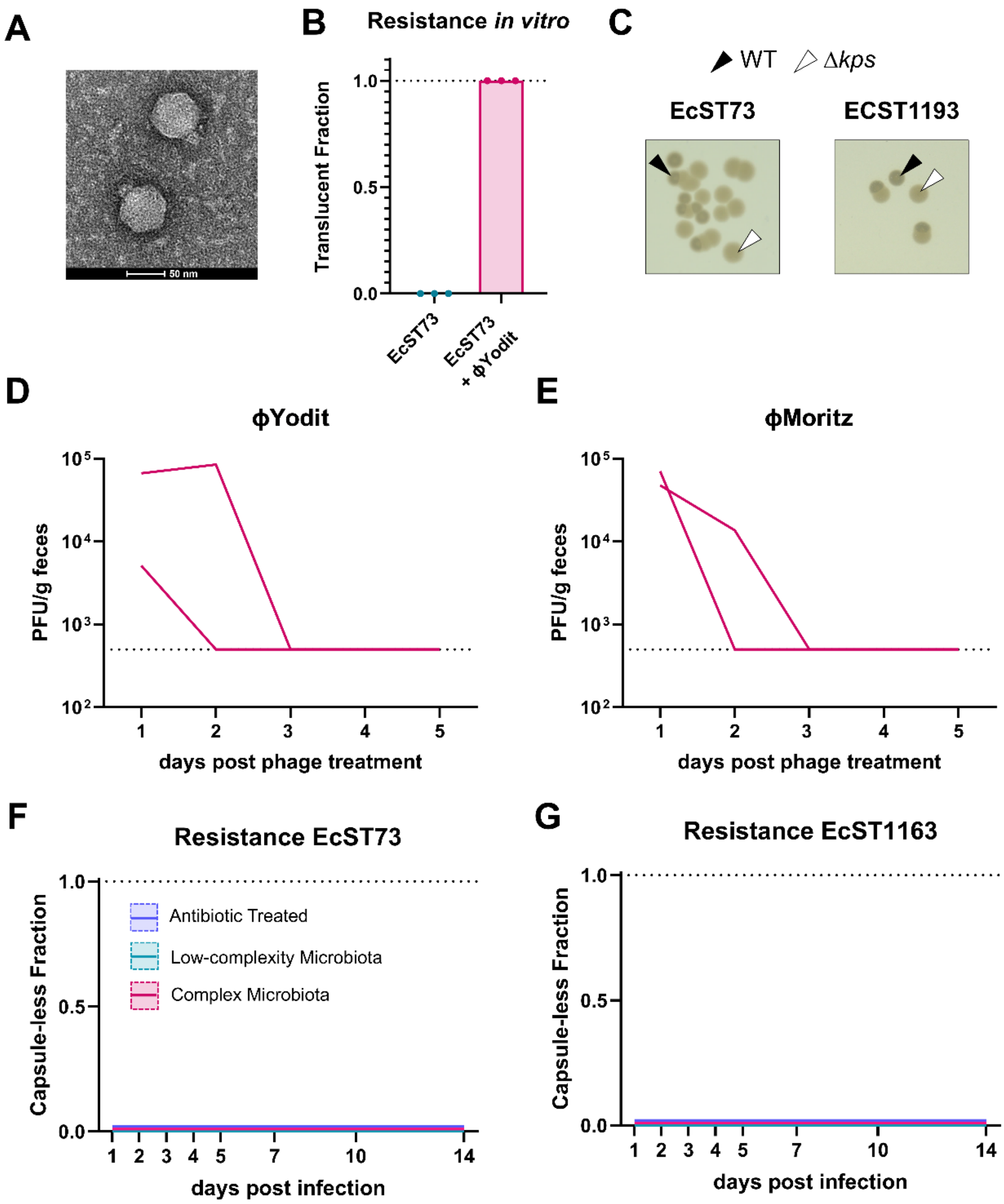

Supplementary Figure 1: Virion morphology, translucent colony morphology and phage titres in uninfected mice. (**A**) Transmission Electron Microscopy image of phage ϕYodit with negative staining. (**B**) Emergence of phage resistance in EcST73 after exposure to phage ϕYodit *in vitro* in liquid culture. (**C**) Example of WT and capsule-less colonies on salt-free LB agar, showing different morphologies. 10^9 CFU of phage (**D**) ϕYodit or (**E**) ϕMoritz were administered by oral gavage to SPF mice (complex microbiota) and phage titres were followed for 5 days in feces. Absence of phage resistance (translucent colonies) in mice colonised with (**F**) EcST73 or (**G**) EcST1193 without phage treatment (From experiment shown in Figure 1).

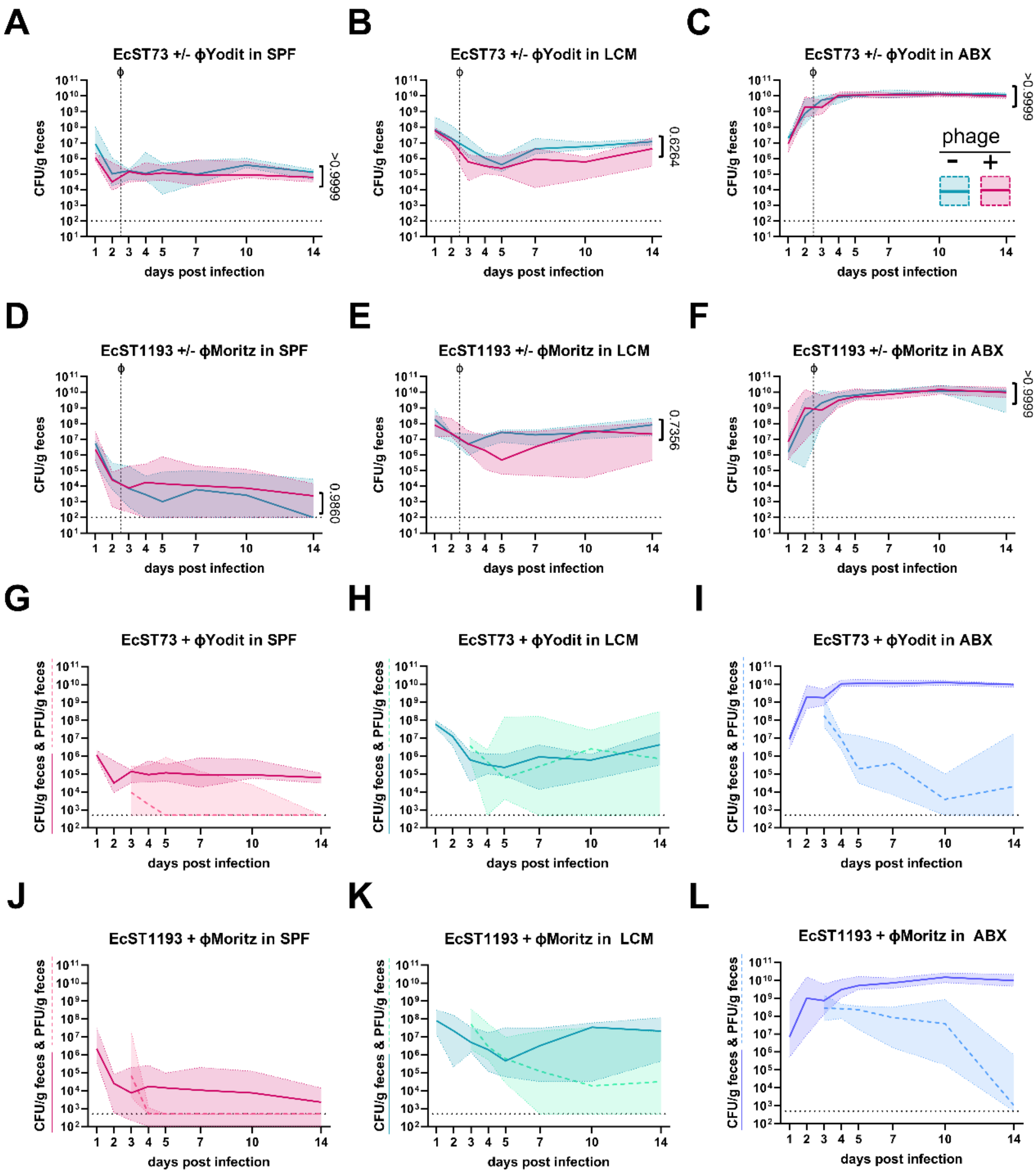

Supplementary Figure 2: Direct comparison between phage-free and phage-treated mice & direct comparison between bacterial counts and phage titres. (**A-F**) Bacterial loads in mice infected with EcST73 or EcST1193, comparison of untreated and phage treated groups (Data shown in Figure 1 and Figure 2). The dashed line indicates the detection limit. CFU: colony-forming units. Lines represent the median and the shaded area is the range. Statistical analysis, calculated using One-way ANOVA of Log-normalised area under the curve (AUC) data, followed by Tukey's multiple comparisons test. P-values are reported. (**G-L**) Bacterial loads and phage titre in phage-treated mice, shown on the same axis. Solid line = bacterial loads, dashed line = phage titre. The dashed line is the detection limit for the phage titre. The detection limit for the bacterial load is 10^2.

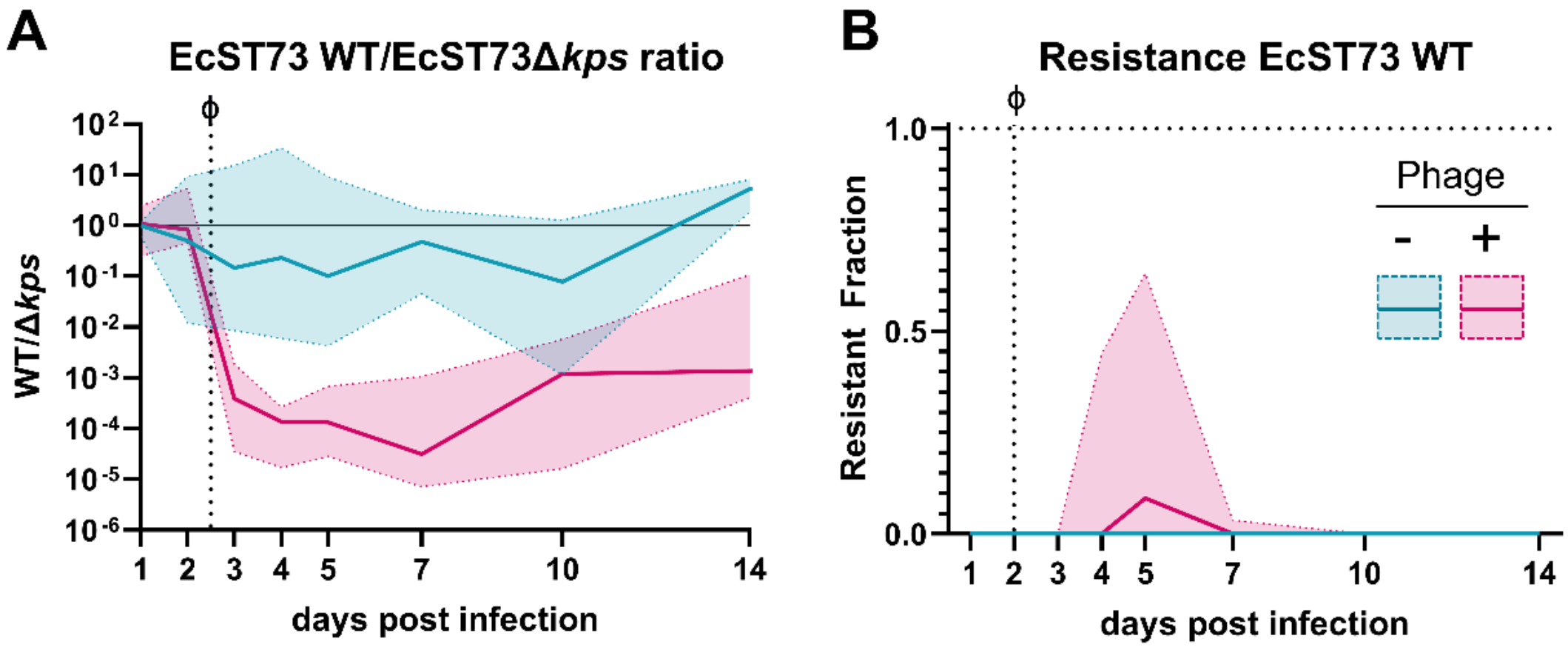

Supplementary Figure 3: Competitive index and phage resistance as measured by colony morphology. Data from the experiment presented in Figure 4. (**A**) Ratio between bacterial levels of WT and capsule-knockout (Δ*kps*) cells. (**B**) Resistant fraction of WT cells as observed by colony morphology. Lines represent the median and the shaded area is the range.

Supplementary Tables

strain background	gene	function	mutation	animal ID	additional mutations
EcST1193 MDBZ1913	*neuS*	polysialic acid synthesis	IS insertion 777 bp	AC193	*malF*
EcST1193 MDBZ1913	*neuE*	polysialic acid synthesis	IS insertion 777 bp	AC194	*malT*
EcST1193 MDBZ1913	*emrR*	regulator of capsule synthesis	SNP EmrR L136R	AC195	*malT, scrA*
EcST1193 MDBZ1913	*emrR*	regulator of capsule synthesis	SNP EmrR L136R	AC196	*malT, scrA*
EcST1193 MDBZ1913	*kpsM*	polysaccharide transport	IS insertion 777 bp	AC197	*malF*
EcST1193 MDBZ1913	*kpsM*	polysaccharide transport	IS insertion 777 bp	AC198	*malF*
EcST73 MDBZ1559	*emrR*	regulator of capsule synthesis	13 bp insertion	AC293	*malI*
EcST73 MDBZ1559	*emrR*	regulator of capsule synthesis	13 bp insertion	AC294	*malI*
EcST73 MDBZ1559	intergenic	polysaccharide transport	1067 bp IS insertion 150 bp upstream of *kpsM*	AC295	*lacC*

Supplementary Table 1: Mutations found in evolved clones. Sequencing of phage-resistant bacterial clones after 14 days of evolution in antibiotic-treated mice exposed to phages ϕYodit or ϕEvar. Sequencing was performed using nanopore technologies long-read sequencing and analysed with the Evo-Genome pipeline.

References

1. Larsson, L., Bertola, A., Wenner, N., Kaur, J., Dion, S., Lemos Rocha, L., Keys, T., Rutschmann, C., Vershynina, K., Dimakou, D., et al. (2026). Phage-steering permits antibody-mediated clearance of *E. coli* K1 from the gut. Nat. Commun. https://doi.org/10.1038/s41467-026-70808-2.

2. Datta, S., Costantino, N., and Court, D.L. (2006). A set of recombineering plasmids for gram-negative bacteria. Gene *379*, 109–115. https://doi.org/10.1016/j.gene.2006.04.018.

3. Lentsch, V., Woller, A., Rocker, A., Aslani, S., Moresi, C., Ruoho, N., Larsson, L., Fattinger, S.A., Wenner, N., Barazzone, E.C., et al. (2025). Vaccine-enhanced competition permits rational bacterial strain replacement in the gut. Science (1979). *388*, 74–81. https://doi.org/10.1126/science.adp5011.

4. Datsenko, K.A., and Wanner, B.L. (2000). One-step inactivation of chromosomal genes in *Escherichia coli* K-12 using PCR products. Proceedings of the National Academy of Sciences *97*, 6640–6645. https://doi.org/10.1073/PNAS.120163297.

5. Kolmogorov, M., Yuan, J., Lin, Y., and Pevzner, P.A. (2019). Assembly of long, error-prone reads using repeat graphs. Nature Biotechnology 2019 37:5 *37*, 540–546. https://doi.org/10.1038/s41587-019-0072-8.

6. Bouras, G., Nepal, R., Houtak, G., Psaltis, A.J., Wormald, P.J., and Vreugde, S. (2023). Pharokka: a fast scalable bacteriophage annotation tool. Bioinformatics *39*. https://doi.org/10.1093/BIOINFORMATICS/BTAC776.

7. Miravet-Verde, S., Cacace, E., Mores, C.R., Rutschmann, C., Lin, C.W., Ruscheweyh, H.J., Cuénod, A., Barazzone, E.C., Marrec, E., Vershynina, K., et al. (2026). In silico typing maps the natural diversity of *Escherichia coli* transporter-dependent capsules. Nat. Microbiol. https://doi.org/10.1038/s41564-026-02323-5.

8. Catalano, C.E. (2018). Bacteriophage lambda: The path from biology to theranostic agent. Wiley Interdiscip. Rev. Nanomed. Nanobiotechnol. *10*. https://doi.org/10.1002/WNAN.1517.

9. McKenzie, G.J., and Craig, N.L. (2006). Fast, easy and efficient: site-specific insertion of transgenes into enterobacterial chromosomes using Tn7 without need for selection of the insertion event. BMC Microbiol. *6*. https://doi.org/10.1186/1471-2180-6-39.

10. Sarma-Rupavtarm, R.B., Ge, Z., Schauer, D.B., Fox, J.G., and Polz, M.F. (2004). Spatial Distribution and Stability of the Eight Microbial Species of the Altered Schaedler Flora in the Mouse Gastrointestinal Tract. Appl. Environ. Microbiol. *70*, 2791. https://doi.org/10.1128/AEM.70.5.2791-2800.2004.

11. Abueg, L.A.L., Afgan, E., Allart, O., Awan, A.H., Bacon, W.A., Baker, D., Bassetti, M., Batut, B., Bernt, M., Blankenberg, D., et al. (2024). The Galaxy platform for accessible, reproducible, and collaborative data analyses: 2024 update. Nucleic Acids Res. *52*, W83–W94. https://doi.org/10.1093/NAR/GKAE410.

12. Wenner, N., Bertola, A., Larsson, L., Rocker, A., Amare Bekele, N., Sauerbeck, C., Lemos Rocha, L.F., Druelle, V., Molari, M., Harms, A., et al. (2025). The intestinal environment potentiates bacteriophage adaptation in response to phenotypic resistance in *Salmonella* Typhimurium. Proceedings of the National Academy of Sciences *122*. https://doi.org/10.1073/PNAS.2507909122.
